# Supplementary material for: Pyruvate Dehydrogenase Kinase 1 inhibition mediated oxidative phosphorylation enhancement in cartilage promotes osteoarthritis progression
Source: BMC Musculoskelet Disord. 2023 Jul 20;24:597. doi: 10.1186/s12891-023-06585-6 (PMC10357736; doi:10.1186/s12891-023-06585-6)
Supplement: Supplementary file 1 — Additional file 1. [file 12891_2023_6585_MOESM1_ESM.zip › BP.Gsea.1653623667859/GOBP_GENERATION_OF_PRECURSOR_METABOLITES_AND_ENERGY.html]

Details for gene set GOBP\_GENERATION\_OF\_PRECURSOR\_METABOLITES\_AND\_ENERGY[GSEA]

|  || Dataset | 55457\_collapsed\_to\_symbols.cls.cls #OA\_versus\_normal.cls.cls #OA\_versus\_normal\_repos |
| Phenotype | cls.cls#OA\_versus\_normal\_repos |
| Upregulated in class | OA |
| GeneSet | GOBP\_GENERATION\_OF\_PRECURSOR\_METABOLITES\_AND\_ENERGY |
| Enrichment Score (ES) | 0.3372165 |
| Normalized Enrichment Score (NES) | 1.9015887 |
| Nominal p-value | 0.0 |
| FDR q-value | 0.044016168 |
| FWER p-Value | 0.752 |
Table: GSEA Results Summary

  

Fig 1: Enrichment plot: GOBP\_GENERATION\_OF\_PRECURSOR\_METABOLITES\_AND\_ENERGY      
 Profile of the Running ES Score & Positions of GeneSet Members on the Rank Ordered List

  

| SYMBOL | TITLE | RANK IN GENE LIST | RANK METRIC SCORE | RUNNING ES | CORE ENRICHMENT || 1 | PPP1R3C | protein phosphatase 1 regulatory subunit 3C [Source:HGNC Symbol;Acc:HGNC:9293] | 41 | 1.008 | 0.0043 | Yes |
| 2 | UQCR10 | "ubiquinol-cytochrome c reductase, complex III subunit X [Source:HGNC Symbol;Acc:HGNC:30863]" | 53 | 0.987 | 0.0107 | Yes |
| 3 | COX17 | cytochrome c oxidase copper chaperone COX17 [Source:HGNC Symbol;Acc:HGNC:2264] | 64 | 0.966 | 0.0172 | Yes |
| 4 | NDUFA8 | NADH:ubiquinone oxidoreductase subunit A8 [Source:HGNC Symbol;Acc:HGNC:7692] | 67 | 0.961 | 0.0242 | Yes |
| 5 | DGUOK | deoxyguanosine kinase [Source:HGNC Symbol;Acc:HGNC:2858] | 96 | 0.917 | 0.0288 | Yes |
| 6 | QDPR | quinoid dihydropteridine reductase [Source:HGNC Symbol;Acc:HGNC:9752] | 112 | 0.897 | 0.0343 | Yes |
| 7 | PRKAG1 | protein kinase AMP-activated non-catalytic subunit gamma 1 [Source:HGNC Symbol;Acc:HGNC:9385] | 138 | 0.870 | 0.0388 | Yes |
| 8 | PDHB | pyruvate dehydrogenase E1 subunit beta [Source:HGNC Symbol;Acc:HGNC:8808] | 149 | 0.862 | 0.0444 | Yes |
| 9 | GPI | glucose-6-phosphate isomerase [Source:HGNC Symbol;Acc:HGNC:4458] | 166 | 0.842 | 0.0494 | Yes |
| 10 | NDUFS3 | NADH:ubiquinone oxidoreductase core subunit S3 [Source:HGNC Symbol;Acc:HGNC:7710] | 177 | 0.836 | 0.0549 | Yes |
| 11 | NDUFA1 | NADH:ubiquinone oxidoreductase subunit A1 [Source:HGNC Symbol;Acc:HGNC:7683] | 194 | 0.826 | 0.0598 | Yes |
| 12 | GHITM | growth hormone inducible transmembrane protein [Source:HGNC Symbol;Acc:HGNC:17281] | 213 | 0.809 | 0.0644 | Yes |
| 13 | PANK2 | pantothenate kinase 2 [Source:HGNC Symbol;Acc:HGNC:15894] | 220 | 0.805 | 0.0699 | Yes |
| 14 | RBKS | ribokinase [Source:HGNC Symbol;Acc:HGNC:30325] | 225 | 0.802 | 0.0756 | Yes |
| 15 | NDUFA13 | NADH:ubiquinone oxidoreductase subunit A13 [Source:HGNC Symbol;Acc:HGNC:17194] | 236 | 0.795 | 0.0807 | Yes |
| 16 | NDUFA10 | NADH:ubiquinone oxidoreductase subunit A10 [Source:HGNC Symbol;Acc:HGNC:7684] | 243 | 0.788 | 0.0861 | Yes |
| 17 | ADPGK | ADP dependent glucokinase [Source:HGNC Symbol;Acc:HGNC:25250] | 249 | 0.785 | 0.0916 | Yes |
| 18 | P4HA2 | prolyl 4-hydroxylase subunit alpha 2 [Source:HGNC Symbol;Acc:HGNC:8547] | 258 | 0.779 | 0.0968 | Yes |
| 19 | NDUFB6 | NADH:ubiquinone oxidoreductase subunit B6 [Source:HGNC Symbol;Acc:HGNC:7701] | 262 | 0.779 | 0.1024 | Yes |
| 20 | MDH1 | malate dehydrogenase 1 [Source:HGNC Symbol;Acc:HGNC:6970] | 271 | 0.772 | 0.1075 | Yes |
| 21 | ACO2 | aconitase 2 [Source:HGNC Symbol;Acc:HGNC:118] | 290 | 0.756 | 0.1117 | Yes |
| 22 | PDIA5 | protein disulfide isomerase family A member 5 [Source:HGNC Symbol;Acc:HGNC:24811] | 324 | 0.735 | 0.1145 | Yes |
| 23 | PGAM1 | phosphoglycerate mutase 1 [Source:HGNC Symbol;Acc:HGNC:8888] | 341 | 0.727 | 0.1187 | Yes |
| 24 | TREX1 | three prime repair exonuclease 1 [Source:HGNC Symbol;Acc:HGNC:12269] | 393 | 0.709 | 0.1199 | Yes |
| 25 | FECH | ferrochelatase [Source:HGNC Symbol;Acc:HGNC:3647] | 396 | 0.707 | 0.1250 | Yes |
| 26 | THTPA | thiamine triphosphatase [Source:HGNC Symbol;Acc:HGNC:18987] | 418 | 0.698 | 0.1286 | Yes |
| 27 | PSEN1 | presenilin 1 [Source:HGNC Symbol;Acc:HGNC:9508] | 425 | 0.695 | 0.1333 | Yes |
| 28 | UQCRC1 | ubiquinol-cytochrome c reductase core protein 1 [Source:HGNC Symbol;Acc:HGNC:12585] | 473 | 0.672 | 0.1345 | Yes |
| 29 | NDUFB3 | NADH:ubiquinone oxidoreductase subunit B3 [Source:HGNC Symbol;Acc:HGNC:7698] | 483 | 0.667 | 0.1388 | Yes |
| 30 | CYBA | cytochrome b-245 alpha chain [Source:HGNC Symbol;Acc:HGNC:2577] | 501 | 0.658 | 0.1423 | Yes |
| 31 | NDUFS4 | NADH:ubiquinone oxidoreductase subunit S4 [Source:HGNC Symbol;Acc:HGNC:7711] | 531 | 0.647 | 0.1449 | Yes |
| 32 | NDUFA3 | NADH:ubiquinone oxidoreductase subunit A3 [Source:HGNC Symbol;Acc:HGNC:7686] | 550 | 0.641 | 0.1482 | Yes |
| 33 | COQ9 | coenzyme Q9 [Source:HGNC Symbol;Acc:HGNC:25302] | 561 | 0.636 | 0.1522 | Yes |
| 34 | ATP5PD | ATP synthase peripheral stalk subunit d [Source:HGNC Symbol;Acc:HGNC:845] | 564 | 0.635 | 0.1568 | Yes |
| 35 | DLD | dihydrolipoamide dehydrogenase [Source:HGNC Symbol;Acc:HGNC:2898] | 565 | 0.635 | 0.1615 | Yes |
| 36 | COX5B | cytochrome c oxidase subunit 5B [Source:HGNC Symbol;Acc:HGNC:2269] | 585 | 0.628 | 0.1647 | Yes |
| 37 | PPP1CA | protein phosphatase 1 catalytic subunit alpha [Source:HGNC Symbol;Acc:HGNC:9281] | 603 | 0.622 | 0.1680 | Yes |
| 38 | MDH2 | malate dehydrogenase 2 [Source:HGNC Symbol;Acc:HGNC:6971] | 615 | 0.618 | 0.1717 | Yes |
| 39 | ANTKMT | adenine nucleotide translocase lysine methyltransferase [Source:HGNC Symbol;Acc:HGNC:14152] | 618 | 0.618 | 0.1761 | Yes |
| 40 | NDUFA4 | NDUFA4 mitochondrial complex associated [Source:HGNC Symbol;Acc:HGNC:7687] | 624 | 0.613 | 0.1803 | Yes |
| 41 | COX6A1 | cytochrome c oxidase subunit 6A1 [Source:HGNC Symbol;Acc:HGNC:2277] | 653 | 0.605 | 0.1826 | Yes |
| 42 | PPP1R3D | protein phosphatase 1 regulatory subunit 3D [Source:HGNC Symbol;Acc:HGNC:9294] | 708 | 0.591 | 0.1827 | Yes |
| 43 | MSH2 | mutS homolog 2 [Source:HGNC Symbol;Acc:HGNC:7325] | 739 | 0.584 | 0.1847 | Yes |
| 44 | PPP1CC | protein phosphatase 1 catalytic subunit gamma [Source:HGNC Symbol;Acc:HGNC:9283] | 744 | 0.583 | 0.1887 | Yes |
| 45 | FBP1 | fructose-bisphosphatase 1 [Source:HGNC Symbol;Acc:HGNC:3606] | 772 | 0.576 | 0.1908 | Yes |
| 46 | ATP5PO | ATP synthase peripheral stalk subunit OSCP [Source:HGNC Symbol;Acc:HGNC:850] | 840 | 0.558 | 0.1896 | Yes |
| 47 | PHKB | phosphorylase kinase regulatory subunit beta [Source:HGNC Symbol;Acc:HGNC:8927] | 853 | 0.554 | 0.1928 | Yes |
| 48 | ALDOC | "aldolase, fructose-bisphosphate C [Source:HGNC Symbol;Acc:HGNC:418]" | 870 | 0.551 | 0.1957 | Yes |
| 49 | NDUFA2 | NADH:ubiquinone oxidoreductase subunit A2 [Source:HGNC Symbol;Acc:HGNC:7685] | 878 | 0.549 | 0.1992 | Yes |
| 50 | NDUFB5 | NADH:ubiquinone oxidoreductase subunit B5 [Source:HGNC Symbol;Acc:HGNC:7700] | 902 | 0.542 | 0.2014 | Yes |
| 51 | TACO1 | translational activator of cytochrome c oxidase I [Source:HGNC Symbol;Acc:HGNC:24316] | 923 | 0.536 | 0.2038 | Yes |
| 52 | NDUFC2 | NADH:ubiquinone oxidoreductase subunit C2 [Source:HGNC Symbol;Acc:HGNC:7706] | 929 | 0.532 | 0.2074 | Yes |
| 53 | NDUFA9 | NADH:ubiquinone oxidoreductase subunit A9 [Source:HGNC Symbol;Acc:HGNC:7693] | 943 | 0.529 | 0.2103 | Yes |
| 54 | DNAJC15 | DnaJ heat shock protein family (Hsp40) member C15 [Source:HGNC Symbol;Acc:HGNC:20325] | 954 | 0.527 | 0.2134 | Yes |
| 55 | IDH2 | isocitrate dehydrogenase (NADP(+)) 2 [Source:HGNC Symbol;Acc:HGNC:5383] | 967 | 0.525 | 0.2164 | Yes |
| 56 | NDUFB4 | NADH:ubiquinone oxidoreductase subunit B4 [Source:HGNC Symbol;Acc:HGNC:7699] | 968 | 0.525 | 0.2203 | Yes |
| 57 | DERA | deoxyribose-phosphate aldolase [Source:HGNC Symbol;Acc:HGNC:24269] | 975 | 0.523 | 0.2237 | Yes |
| 58 | PGLS | 6-phosphogluconolactonase [Source:HGNC Symbol;Acc:HGNC:8903] | 976 | 0.523 | 0.2277 | Yes |
| 59 | UQCRQ | ubiquinol-cytochrome c reductase complex III subunit VII [Source:HGNC Symbol;Acc:HGNC:29594] | 986 | 0.521 | 0.2308 | Yes |
| 60 | ALDH2 | aldehyde dehydrogenase 2 family member [Source:HGNC Symbol;Acc:HGNC:404] | 1003 | 0.517 | 0.2334 | Yes |
| 61 | ACAT1 | acetyl-CoA acetyltransferase 1 [Source:HGNC Symbol;Acc:HGNC:93] | 1017 | 0.513 | 0.2362 | Yes |
| 62 | NDUFB7 | NADH:ubiquinone oxidoreductase subunit B7 [Source:HGNC Symbol;Acc:HGNC:7702] | 1033 | 0.510 | 0.2388 | Yes |
| 63 | NDUFB1 | NADH:ubiquinone oxidoreductase subunit B1 [Source:HGNC Symbol;Acc:HGNC:7695] | 1055 | 0.506 | 0.2409 | Yes |
| 64 | NDUFS7 | NADH:ubiquinone oxidoreductase core subunit S7 [Source:HGNC Symbol;Acc:HGNC:7714] | 1071 | 0.501 | 0.2435 | Yes |
| 65 | NDUFC1 | NADH:ubiquinone oxidoreductase subunit C1 [Source:HGNC Symbol;Acc:HGNC:7705] | 1091 | 0.499 | 0.2457 | Yes |
| 66 | NDUFA7 | NADH:ubiquinone oxidoreductase subunit A7 [Source:HGNC Symbol;Acc:HGNC:7691] | 1097 | 0.496 | 0.2490 | Yes |
| 67 | COX8A | cytochrome c oxidase subunit 8A [Source:HGNC Symbol;Acc:HGNC:2294] | 1105 | 0.494 | 0.2521 | Yes |
| 68 | CAVIN3 | caveolae associated protein 3 [Source:HGNC Symbol;Acc:HGNC:9400] | 1141 | 0.488 | 0.2529 | Yes |
| 69 | GAA | alpha glucosidase [Source:HGNC Symbol;Acc:HGNC:4065] | 1149 | 0.486 | 0.2560 | Yes |
| 70 | FDX1 | ferredoxin 1 [Source:HGNC Symbol;Acc:HGNC:3638] | 1168 | 0.483 | 0.2582 | Yes |
| 71 | ARL2 | ADP ribosylation factor like GTPase 2 [Source:HGNC Symbol;Acc:HGNC:693] | 1179 | 0.481 | 0.2610 | Yes |
| 72 | AKT1 | AKT serine/threonine kinase 1 [Source:HGNC Symbol;Acc:HGNC:391] | 1192 | 0.478 | 0.2636 | Yes |
| 73 | NDUFS8 | NADH:ubiquinone oxidoreductase core subunit S8 [Source:HGNC Symbol;Acc:HGNC:7715] | 1208 | 0.474 | 0.2659 | Yes |
| 74 | GYG1 | glycogenin 1 [Source:HGNC Symbol;Acc:HGNC:4699] | 1209 | 0.474 | 0.2695 | Yes |
| 75 | NDUFS1 | NADH:ubiquinone oxidoreductase core subunit S1 [Source:HGNC Symbol;Acc:HGNC:7707] | 1229 | 0.471 | 0.2715 | Yes |
| 76 | ETFB | electron transfer flavoprotein subunit beta [Source:HGNC Symbol;Acc:HGNC:3482] | 1289 | 0.460 | 0.2702 | Yes |
| 77 | ATP5PB | ATP synthase peripheral stalk-membrane subunit b [Source:HGNC Symbol;Acc:HGNC:840] | 1293 | 0.459 | 0.2734 | Yes |
| 78 | ATP5MF | ATP synthase membrane subunit f [Source:HGNC Symbol;Acc:HGNC:848] | 1330 | 0.453 | 0.2739 | Yes |
| 79 | ACOX1 | acyl-CoA oxidase 1 [Source:HGNC Symbol;Acc:HGNC:119] | 1401 | 0.443 | 0.2716 | Yes |
| 80 | PARK7 | Parkinsonism associated deglycase [Source:HGNC Symbol;Acc:HGNC:16369] | 1406 | 0.442 | 0.2746 | Yes |
| 81 | NDUFS6 | NADH:ubiquinone oxidoreductase subunit S6 [Source:HGNC Symbol;Acc:HGNC:7713] | 1417 | 0.441 | 0.2771 | Yes |
| 82 | CYB561 | cytochrome b561 [Source:HGNC Symbol;Acc:HGNC:2571] | 1459 | 0.435 | 0.2770 | Yes |
| 83 | SUCLG2 | succinate-CoA ligase GDP-forming subunit beta [Source:HGNC Symbol;Acc:HGNC:11450] | 1472 | 0.432 | 0.2793 | Yes |
| 84 | SHMT2 | serine hydroxymethyltransferase 2 [Source:HGNC Symbol;Acc:HGNC:10852] | 1518 | 0.426 | 0.2789 | Yes |
| 85 | ISCU | iron-sulfur cluster assembly enzyme [Source:HGNC Symbol;Acc:HGNC:29882] | 1530 | 0.423 | 0.2812 | Yes |
| 86 | FDXR | ferredoxin reductase [Source:HGNC Symbol;Acc:HGNC:3642] | 1536 | 0.423 | 0.2839 | Yes |
| 87 | NOX4 | NADPH oxidase 4 [Source:HGNC Symbol;Acc:HGNC:7891] | 1563 | 0.418 | 0.2850 | Yes |
| 88 | NQO2 | N-ribosyldihydronicotinamide:quinone reductase 2 [Source:HGNC Symbol;Acc:HGNC:7856] | 1568 | 0.418 | 0.2878 | Yes |
| 89 | ATP5IF1 | ATP synthase inhibitory factor subunit 1 [Source:HGNC Symbol;Acc:HGNC:871] | 1575 | 0.417 | 0.2904 | Yes |
| 90 | COX11 | cytochrome c oxidase copper chaperone COX11 [Source:HGNC Symbol;Acc:HGNC:2261] | 1622 | 0.410 | 0.2898 | Yes |
| 91 | NDUFV2 | NADH:ubiquinone oxidoreductase core subunit V2 [Source:HGNC Symbol;Acc:HGNC:7717] | 1642 | 0.407 | 0.2913 | Yes |
| 92 | NDUFV1 | NADH:ubiquinone oxidoreductase core subunit V1 [Source:HGNC Symbol;Acc:HGNC:7716] | 1663 | 0.404 | 0.2927 | Yes |
| 93 | HMGCL | 3-hydroxy-3-methylglutaryl-CoA lyase [Source:HGNC Symbol;Acc:HGNC:5005] | 1703 | 0.398 | 0.2926 | Yes |
| 94 | CAT | catalase [Source:HGNC Symbol;Acc:HGNC:1516] | 1731 | 0.394 | 0.2934 | Yes |
| 95 | ME1 | malic enzyme 1 [Source:HGNC Symbol;Acc:HGNC:6983] | 1746 | 0.392 | 0.2952 | Yes |
| 96 | CEBPA | CCAAT enhancer binding protein alpha [Source:HGNC Symbol;Acc:HGNC:1833] | 1750 | 0.392 | 0.2979 | Yes |
| 97 | GNPDA1 | glucosamine-6-phosphate deaminase 1 [Source:HGNC Symbol;Acc:HGNC:4417] | 1795 | 0.385 | 0.2972 | Yes |
| 98 | ETFA | electron transfer flavoprotein subunit alpha [Source:HGNC Symbol;Acc:HGNC:3481] | 1797 | 0.385 | 0.3000 | Yes |
| 99 | SNCA | synuclein alpha [Source:HGNC Symbol;Acc:HGNC:11138] | 1800 | 0.384 | 0.3027 | Yes |
| 100 | SDHB | succinate dehydrogenase complex iron sulfur subunit B [Source:HGNC Symbol;Acc:HGNC:10681] | 1818 | 0.382 | 0.3042 | Yes |
| 101 | STOML2 | stomatin like 2 [Source:HGNC Symbol;Acc:HGNC:14559] | 1819 | 0.382 | 0.3071 | Yes |
| 102 | SURF1 | SURF1 cytochrome c oxidase assembly factor [Source:HGNC Symbol;Acc:HGNC:11474] | 1829 | 0.381 | 0.3092 | Yes |
| 103 | HTR2A | 5-hydroxytryptamine receptor 2A [Source:HGNC Symbol;Acc:HGNC:5293] | 1847 | 0.379 | 0.3107 | Yes |
| 104 | ME2 | malic enzyme 2 [Source:HGNC Symbol;Acc:HGNC:6984] | 1870 | 0.377 | 0.3117 | Yes |
| 105 | NIPSNAP2 | nipsnap homolog 2 [Source:HGNC Symbol;Acc:HGNC:4179] | 1886 | 0.374 | 0.3133 | Yes |
| 106 | HMGCS2 | 3-hydroxy-3-methylglutaryl-CoA synthase 2 [Source:HGNC Symbol;Acc:HGNC:5008] | 1888 | 0.374 | 0.3160 | Yes |
| 107 | PYGM | "glycogen phosphorylase, muscle associated [Source:HGNC Symbol;Acc:HGNC:9726]" | 1952 | 0.367 | 0.3137 | Yes |
| 108 | SRD5A1 | steroid 5 alpha-reductase 1 [Source:HGNC Symbol;Acc:HGNC:11284] | 1973 | 0.364 | 0.3148 | Yes |
| 109 | COX7A1 | cytochrome c oxidase subunit 7A1 [Source:HGNC Symbol;Acc:HGNC:2287] | 2045 | 0.356 | 0.3118 | Yes |
| 110 | PINK1 | PTEN induced kinase 1 [Source:HGNC Symbol;Acc:HGNC:14581] | 2097 | 0.350 | 0.3103 | Yes |
| 111 | TNF | tumor necrosis factor [Source:HGNC Symbol;Acc:HGNC:11892] | 2098 | 0.350 | 0.3130 | Yes |
| 112 | COX4I1 | cytochrome c oxidase subunit 4I1 [Source:HGNC Symbol;Acc:HGNC:2265] | 2167 | 0.342 | 0.3101 | Yes |
| 113 | ATP5F1C | ATP synthase F1 subunit gamma [Source:HGNC Symbol;Acc:HGNC:833] | 2190 | 0.339 | 0.3108 | Yes |
| 114 | MAOB | monoamine oxidase B [Source:HGNC Symbol;Acc:HGNC:6834] | 2192 | 0.339 | 0.3133 | Yes |
| 115 | NCF1 | neutrophil cytosolic factor 1 [Source:HGNC Symbol;Acc:HGNC:7660] | 2195 | 0.339 | 0.3157 | Yes |
| 116 | IREB2 | iron responsive element binding protein 2 [Source:HGNC Symbol;Acc:HGNC:6115] | 2215 | 0.337 | 0.3167 | Yes |
| 117 | SDHA | succinate dehydrogenase complex flavoprotein subunit A [Source:HGNC Symbol;Acc:HGNC:10680] | 2231 | 0.335 | 0.3180 | Yes |
| 118 | PFKM | "phosphofructokinase, muscle [Source:HGNC Symbol;Acc:HGNC:8877]" | 2260 | 0.331 | 0.3182 | Yes |
| 119 | NDUFS5 | NADH:ubiquinone oxidoreductase subunit S5 [Source:HGNC Symbol;Acc:HGNC:7712] | 2267 | 0.330 | 0.3202 | Yes |
| 120 | SUCLG1 | succinate-CoA ligase GDP/ADP-forming subunit alpha [Source:HGNC Symbol;Acc:HGNC:11449] | 2269 | 0.330 | 0.3226 | Yes |
| 121 | PPP1R2 | protein phosphatase 1 regulatory inhibitor subunit 2 [Source:HGNC Symbol;Acc:HGNC:9288] | 2277 | 0.329 | 0.3245 | Yes |
| 122 | IGF1 | insulin like growth factor 1 [Source:HGNC Symbol;Acc:HGNC:5464] | 2286 | 0.328 | 0.3263 | Yes |
| 123 | CCNB1 | cyclin B1 [Source:HGNC Symbol;Acc:HGNC:1579] | 2359 | 0.319 | 0.3229 | Yes |
| 124 | BPGM | bisphosphoglycerate mutase [Source:HGNC Symbol;Acc:HGNC:1093] | 2398 | 0.315 | 0.3222 | Yes |
| 125 | PYGB | glycogen phosphorylase B [Source:HGNC Symbol;Acc:HGNC:9723] | 2400 | 0.315 | 0.3245 | Yes |
| 126 | NNT | nicotinamide nucleotide transhydrogenase [Source:HGNC Symbol;Acc:HGNC:7863] | 2425 | 0.311 | 0.3249 | Yes |
| 127 | CHCHD2 | coiled-coil-helix-coiled-coil-helix domain containing 2 [Source:HGNC Symbol;Acc:HGNC:21645] | 2431 | 0.310 | 0.3268 | Yes |
| 128 | PDHA1 | pyruvate dehydrogenase E1 subunit alpha 1 [Source:HGNC Symbol;Acc:HGNC:8806] | 2433 | 0.310 | 0.3291 | Yes |
| 129 | IL10RB | interleukin 10 receptor subunit beta [Source:HGNC Symbol;Acc:HGNC:5965] | 2438 | 0.309 | 0.3310 | Yes |
| 130 | NDUFB2 | NADH:ubiquinone oxidoreductase subunit B2 [Source:HGNC Symbol;Acc:HGNC:7697] | 2460 | 0.307 | 0.3317 | Yes |
| 131 | SPHK2 | sphingosine kinase 2 [Source:HGNC Symbol;Acc:HGNC:18859] | 2478 | 0.305 | 0.3326 | Yes |
| 132 | COX7A2L | cytochrome c oxidase subunit 7A2 like [Source:HGNC Symbol;Acc:HGNC:2289] | 2517 | 0.302 | 0.3318 | Yes |
| 133 | NCOR1 | nuclear receptor corepressor 1 [Source:HGNC Symbol;Acc:HGNC:7672] | 2549 | 0.297 | 0.3315 | Yes |
| 134 | PGK2 | phosphoglycerate kinase 2 [Source:HGNC Symbol;Acc:HGNC:8898] | 2566 | 0.295 | 0.3324 | Yes |
| 135 | CROT | carnitine O-octanoyltransferase [Source:HGNC Symbol;Acc:HGNC:2366] | 2567 | 0.295 | 0.3346 | Yes |
| 136 | TIGAR | TP53 induced glycolysis regulatory phosphatase [Source:HGNC Symbol;Acc:HGNC:1185] | 2580 | 0.293 | 0.3359 | Yes |
| 137 | ASIP | agouti signaling protein [Source:HGNC Symbol;Acc:HGNC:745] | 2596 | 0.291 | 0.3368 | Yes |
| 138 | NDUFB8 | NADH:ubiquinone oxidoreductase subunit B8 [Source:HGNC Symbol;Acc:HGNC:7703] | 2623 | 0.288 | 0.3369 | Yes |
| 139 | BLOC1S1 | biogenesis of lysosomal organelles complex 1 subunit 1 [Source:HGNC Symbol;Acc:HGNC:4200] | 2657 | 0.284 | 0.3364 | Yes |
| 140 | AKR7A2 | aldo-keto reductase family 7 member A2 [Source:HGNC Symbol;Acc:HGNC:389] | 2690 | 0.280 | 0.3359 | Yes |
| 141 | ATP5F1D | ATP synthase F1 subunit delta [Source:HGNC Symbol;Acc:HGNC:837] | 2701 | 0.279 | 0.3372 | Yes |
| 142 | PPP1R3A | protein phosphatase 1 regulatory subunit 3A [Source:HGNC Symbol;Acc:HGNC:9291] | 2732 | 0.277 | 0.3369 | No |
| 143 | KHK | ketohexokinase [Source:HGNC Symbol;Acc:HGNC:6315] | 2809 | 0.269 | 0.3328 | No |
| 144 | OXCT1 | 3-oxoacid CoA-transferase 1 [Source:HGNC Symbol;Acc:HGNC:8527] | 2897 | 0.261 | 0.3278 | No |
| 145 | ATP5MG | ATP synthase membrane subunit g [Source:HGNC Symbol;Acc:HGNC:14247] | 2941 | 0.256 | 0.3263 | No |
| 146 | OPN3 | opsin 3 [Source:HGNC Symbol;Acc:HGNC:14007] | 2961 | 0.254 | 0.3266 | No |
| 147 | HSD17B6 | hydroxysteroid 17-beta dehydrogenase 6 [Source:HGNC Symbol;Acc:HGNC:23316] | 2965 | 0.254 | 0.3283 | No |
| 148 | PGAM2 | phosphoglycerate mutase 2 [Source:HGNC Symbol;Acc:HGNC:8889] | 3008 | 0.249 | 0.3268 | No |
| 149 | ATP5F1B | ATP synthase F1 subunit beta [Source:HGNC Symbol;Acc:HGNC:830] | 3024 | 0.247 | 0.3274 | No |
| 150 | NDUFS2 | NADH:ubiquinone oxidoreductase core subunit S2 [Source:HGNC Symbol;Acc:HGNC:7708] | 3072 | 0.243 | 0.3255 | No |
| 151 | PCDH12 | protocadherin 12 [Source:HGNC Symbol;Acc:HGNC:8657] | 3081 | 0.242 | 0.3267 | No |
| 152 | DHTKD1 | dehydrogenase E1 and transketolase domain containing 1 [Source:HGNC Symbol;Acc:HGNC:23537] | 3112 | 0.239 | 0.3260 | No |
| 153 | AKR1B1 | aldo-keto reductase family 1 member B [Source:HGNC Symbol;Acc:HGNC:381] | 3138 | 0.236 | 0.3258 | No |
| 154 | SDHC | succinate dehydrogenase complex subunit C [Source:HGNC Symbol;Acc:HGNC:10682] | 3145 | 0.235 | 0.3271 | No |
| 155 | RPE | ribulose-5-phosphate-3-epimerase [Source:HGNC Symbol;Acc:HGNC:10293] | 3153 | 0.235 | 0.3283 | No |
| 156 | COX7A2 | cytochrome c oxidase subunit 7A2 [Source:HGNC Symbol;Acc:HGNC:2288] | 3164 | 0.234 | 0.3292 | No |
| 157 | ENO3 | enolase 3 [Source:HGNC Symbol;Acc:HGNC:3354] | 3207 | 0.230 | 0.3276 | No |
| 158 | HAAO | "3-hydroxyanthranilate 3,4-dioxygenase [Source:HGNC Symbol;Acc:HGNC:4796]" | 3209 | 0.229 | 0.3292 | No |
| 159 | SUCLA2 | succinate-CoA ligase ADP-forming subunit beta [Source:HGNC Symbol;Acc:HGNC:11448] | 3228 | 0.228 | 0.3295 | No |
| 160 | SLC4A1 | solute carrier family 4 member 1 (Diego blood group) [Source:HGNC Symbol;Acc:HGNC:11027] | 3233 | 0.227 | 0.3309 | No |
| 161 | PID1 | phosphotyrosine interaction domain containing 1 [Source:HGNC Symbol;Acc:HGNC:26084] | 3280 | 0.222 | 0.3288 | No |
| 162 | GBA | glucosylceramidase beta [Source:HGNC Symbol;Acc:HGNC:4177] | 3327 | 0.219 | 0.3268 | No |
| 163 | ENPP1 | ectonucleotide pyrophosphatase/phosphodiesterase 1 [Source:HGNC Symbol;Acc:HGNC:3356] | 3392 | 0.212 | 0.3232 | No |
| 164 | GAPDHS | "glyceraldehyde-3-phosphate dehydrogenase, spermatogenic [Source:HGNC Symbol;Acc:HGNC:24864]" | 3466 | 0.205 | 0.3189 | No |
| 165 | VCP | valosin containing protein [Source:HGNC Symbol;Acc:HGNC:12666] | 3518 | 0.201 | 0.3164 | No |
| 166 | SLC27A5 | solute carrier family 27 member 5 [Source:HGNC Symbol;Acc:HGNC:10999] | 3525 | 0.200 | 0.3174 | No |
| 167 | AGL | "amylo-alpha-1, 6-glucosidase, 4-alpha-glucanotransferase [Source:HGNC Symbol;Acc:HGNC:321]" | 3537 | 0.199 | 0.3180 | No |
| 168 | SHPK | sedoheptulokinase [Source:HGNC Symbol;Acc:HGNC:1492] | 3542 | 0.199 | 0.3191 | No |
| 169 | PFKFB2 | "6-phosphofructo-2-kinase/fructose-2,6-biphosphatase 2 [Source:HGNC Symbol;Acc:HGNC:8873]" | 3547 | 0.198 | 0.3203 | No |
| 170 | TYRP1 | tyrosinase related protein 1 [Source:HGNC Symbol;Acc:HGNC:12450] | 3555 | 0.198 | 0.3212 | No |
| 171 | IFNAR1 | interferon alpha and beta receptor subunit 1 [Source:HGNC Symbol;Acc:HGNC:5432] | 3587 | 0.195 | 0.3202 | No |
| 172 | PASK | PAS domain containing serine/threonine kinase [Source:HGNC Symbol;Acc:HGNC:17270] | 3604 | 0.193 | 0.3204 | No |
| 173 | PTH | parathyroid hormone [Source:HGNC Symbol;Acc:HGNC:9606] | 3652 | 0.190 | 0.3180 | No |
| 174 | COX15 | cytochrome c oxidase assembly homolog COX15 [Source:HGNC Symbol;Acc:HGNC:2263] | 3727 | 0.183 | 0.3135 | No |
| 175 | CDK1 | cyclin dependent kinase 1 [Source:HGNC Symbol;Acc:HGNC:1722] | 3744 | 0.182 | 0.3135 | No |
| 176 | ENOX2 | ecto-NOX disulfide-thiol exchanger 2 [Source:HGNC Symbol;Acc:HGNC:2259] | 3769 | 0.179 | 0.3130 | No |
| 177 | HK3 | hexokinase 3 [Source:HGNC Symbol;Acc:HGNC:4925] | 3792 | 0.178 | 0.3125 | No |
| 178 | PYGL | glycogen phosphorylase L [Source:HGNC Symbol;Acc:HGNC:9725] | 3826 | 0.174 | 0.3112 | No |
| 179 | UQCRH | ubiquinol-cytochrome c reductase hinge protein [Source:HGNC Symbol;Acc:HGNC:12590] | 3827 | 0.174 | 0.3125 | No |
| 180 | NDUFA6 | NADH:ubiquinone oxidoreductase subunit A6 [Source:HGNC Symbol;Acc:HGNC:7690] | 3834 | 0.173 | 0.3133 | No |
| 181 | INS | insulin [Source:HGNC Symbol;Acc:HGNC:6081] | 3885 | 0.166 | 0.3105 | No |
| 182 | GLDC | glycine decarboxylase [Source:HGNC Symbol;Acc:HGNC:4313] | 3913 | 0.163 | 0.3096 | No |
| 183 | PKM | pyruvate kinase M1/2 [Source:HGNC Symbol;Acc:HGNC:9021] | 3963 | 0.159 | 0.3068 | No |
| 184 | PGM1 | phosphoglucomutase 1 [Source:HGNC Symbol;Acc:HGNC:8905] | 3990 | 0.157 | 0.3059 | No |
| 185 | SIRT3 | sirtuin 3 [Source:HGNC Symbol;Acc:HGNC:14931] | 4009 | 0.155 | 0.3057 | No |
| 186 | UQCRC2 | ubiquinol-cytochrome c reductase core protein 2 [Source:HGNC Symbol;Acc:HGNC:12586] | 4020 | 0.154 | 0.3060 | No |
| 187 | OXCT2 | 3-oxoacid CoA-transferase 2 [Source:HGNC Symbol;Acc:HGNC:18606] | 4035 | 0.152 | 0.3060 | No |
| 188 | COX6C | cytochrome c oxidase subunit 6C [Source:HGNC Symbol;Acc:HGNC:2285] | 4036 | 0.152 | 0.3072 | No |
| 189 | SDHD | succinate dehydrogenase complex subunit D [Source:HGNC Symbol;Acc:HGNC:10683] | 4055 | 0.151 | 0.3069 | No |
| 190 | TPI1 | triosephosphate isomerase 1 [Source:HGNC Symbol;Acc:HGNC:12009] | 4059 | 0.150 | 0.3077 | No |
| 191 | CISD1 | CDGSH iron sulfur domain 1 [Source:HGNC Symbol;Acc:HGNC:30880] | 4104 | 0.146 | 0.3053 | No |
| 192 | ATP5F1A | ATP synthase F1 subunit alpha [Source:HGNC Symbol;Acc:HGNC:823] | 4137 | 0.143 | 0.3038 | No |
| 193 | CIAPIN1 | cytokine induced apoptosis inhibitor 1 [Source:HGNC Symbol;Acc:HGNC:28050] | 4161 | 0.141 | 0.3030 | No |
| 194 | ALDOA | "aldolase, fructose-bisphosphate A [Source:HGNC Symbol;Acc:HGNC:414]" | 4289 | 0.129 | 0.2938 | No |
| 195 | CYB5R4 | cytochrome b5 reductase 4 [Source:HGNC Symbol;Acc:HGNC:20147] | 4348 | 0.124 | 0.2901 | No |
| 196 | PHB2 | prohibitin 2 [Source:HGNC Symbol;Acc:HGNC:30306] | 4354 | 0.124 | 0.2906 | No |
| 197 | PRKAG2 | protein kinase AMP-activated non-catalytic subunit gamma 2 [Source:HGNC Symbol;Acc:HGNC:9386] | 4362 | 0.123 | 0.2910 | No |
| 198 | IGF2 | insulin like growth factor 2 [Source:HGNC Symbol;Acc:HGNC:5466] | 4378 | 0.123 | 0.2907 | No |
| 199 | NDUFAF1 | NADH:ubiquinone oxidoreductase complex assembly factor 1 [Source:HGNC Symbol;Acc:HGNC:18828] | 4388 | 0.122 | 0.2909 | No |
| 200 | P2RX7 | purinergic receptor P2X 7 [Source:HGNC Symbol;Acc:HGNC:8537] | 4415 | 0.120 | 0.2897 | No |
| 201 | NDUFAB1 | NADH:ubiquinone oxidoreductase subunit AB1 [Source:HGNC Symbol;Acc:HGNC:7694] | 4445 | 0.118 | 0.2883 | No |
| 202 | TEFM | "transcription elongation factor, mitochondrial [Source:HGNC Symbol;Acc:HGNC:26223]" | 4447 | 0.117 | 0.2891 | No |
| 203 | SLC2A6 | solute carrier family 2 member 6 [Source:HGNC Symbol;Acc:HGNC:11011] | 4449 | 0.117 | 0.2899 | No |
| 204 | IDH3G | isocitrate dehydrogenase (NAD(+)) 3 non-catalytic subunit gamma [Source:HGNC Symbol;Acc:HGNC:5386] | 4504 | 0.113 | 0.2864 | No |
| 205 | COX7C | cytochrome c oxidase subunit 7C [Source:HGNC Symbol;Acc:HGNC:2292] | 4539 | 0.110 | 0.2845 | No |
| 206 | GSR | glutathione-disulfide reductase [Source:HGNC Symbol;Acc:HGNC:4623] | 4597 | 0.105 | 0.2807 | No |
| 207 | ME3 | malic enzyme 3 [Source:HGNC Symbol;Acc:HGNC:6985] | 4613 | 0.104 | 0.2803 | No |
| 208 | ADH5 | "alcohol dehydrogenase 5 (class III), chi polypeptide [Source:HGNC Symbol;Acc:HGNC:253]" | 4720 | 0.094 | 0.2725 | No |
| 209 | TRAP1 | TNF receptor associated protein 1 [Source:HGNC Symbol;Acc:HGNC:16264] | 4756 | 0.090 | 0.2704 | No |
| 210 | DHRS3 | dehydrogenase/reductase 3 [Source:HGNC Symbol;Acc:HGNC:17693] | 4770 | 0.089 | 0.2700 | No |
| 211 | ATP7A | ATPase copper transporting alpha [Source:HGNC Symbol;Acc:HGNC:869] | 4814 | 0.086 | 0.2672 | No |
| 212 | CYC1 | cytochrome c1 [Source:HGNC Symbol;Acc:HGNC:2579] | 4879 | 0.082 | 0.2627 | No |
| 213 | KL | klotho [Source:HGNC Symbol;Acc:HGNC:6344] | 4901 | 0.080 | 0.2616 | No |
| 214 | ADH1C | "alcohol dehydrogenase 1C (class I), gamma polypeptide [Source:HGNC Symbol;Acc:HGNC:251]" | 4908 | 0.079 | 0.2617 | No |
| 215 | UQCR11 | "ubiquinol-cytochrome c reductase, complex III subunit XI [Source:HGNC Symbol;Acc:HGNC:30862]" | 4917 | 0.078 | 0.2617 | No |
| 216 | ACADM | acyl-CoA dehydrogenase medium chain [Source:HGNC Symbol;Acc:HGNC:89] | 4983 | 0.074 | 0.2570 | No |
| 217 | UQCRB | ubiquinol-cytochrome c reductase binding protein [Source:HGNC Symbol;Acc:HGNC:12582] | 5003 | 0.073 | 0.2560 | No |
| 218 | SLC4A4 | solute carrier family 4 member 4 [Source:HGNC Symbol;Acc:HGNC:11030] | 5027 | 0.071 | 0.2547 | No |
| 219 | SLC25A4 | solute carrier family 25 member 4 [Source:HGNC Symbol;Acc:HGNC:10990] | 5073 | 0.067 | 0.2516 | No |
| 220 | TALDO1 | transaldolase 1 [Source:HGNC Symbol;Acc:HGNC:11559] | 5184 | 0.057 | 0.2433 | No |
| 221 | AKR7A3 | aldo-keto reductase family 7 member A3 [Source:HGNC Symbol;Acc:HGNC:390] | 5192 | 0.057 | 0.2431 | No |
| 222 | DYRK2 | dual specificity tyrosine phosphorylation regulated kinase 2 [Source:HGNC Symbol;Acc:HGNC:3093] | 5224 | 0.054 | 0.2410 | No |
| 223 | COX6A2 | cytochrome c oxidase subunit 6A2 [Source:HGNC Symbol;Acc:HGNC:2279] | 5242 | 0.053 | 0.2401 | No |
| 224 | COX7A2P2 | cytochrome c oxidase subunit 7A2 pseudogene 2 [Source:HGNC Symbol;Acc:HGNC:2290] | 5263 | 0.051 | 0.2389 | No |
| 225 | COX5A | cytochrome c oxidase subunit 5A [Source:HGNC Symbol;Acc:HGNC:2267] | 5329 | 0.045 | 0.2340 | No |
| 226 | SLC25A14 | solute carrier family 25 member 14 [Source:HGNC Symbol;Acc:HGNC:10984] | 5333 | 0.045 | 0.2341 | No |
| 227 | G6PC1 | glucose-6-phosphatase catalytic subunit 1 [Source:HGNC Symbol;Acc:HGNC:4056] | 5389 | 0.041 | 0.2300 | No |
| 228 | IDE | insulin degrading enzyme [Source:HGNC Symbol;Acc:HGNC:5381] | 5491 | 0.033 | 0.2222 | No |
| 229 | PFKP | "phosphofructokinase, platelet [Source:HGNC Symbol;Acc:HGNC:8878]" | 5500 | 0.032 | 0.2218 | No |
| 230 | BNIP3 | BCL2 interacting protein 3 [Source:HGNC Symbol;Acc:HGNC:1084] | 5501 | 0.032 | 0.2220 | No |
| 231 | SLC25A12 | solute carrier family 25 member 12 [Source:HGNC Symbol;Acc:HGNC:10982] | 5506 | 0.032 | 0.2219 | No |
| 232 | UQCRFS1 | "ubiquinol-cytochrome c reductase, Rieske iron-sulfur polypeptide 1 [Source:HGNC Symbol;Acc:HGNC:12587]" | 5509 | 0.032 | 0.2220 | No |
| 233 | TP53 | tumor protein p53 [Source:HGNC Symbol;Acc:HGNC:11998] | 5511 | 0.032 | 0.2221 | No |
| 234 | ENO2 | enolase 2 [Source:HGNC Symbol;Acc:HGNC:3353] | 5572 | 0.026 | 0.2175 | No |
| 235 | NUPR1 | "nuclear protein 1, transcriptional regulator [Source:HGNC Symbol;Acc:HGNC:29990]" | 5616 | 0.023 | 0.2143 | No |
| 236 | PFKL | "phosphofructokinase, liver type [Source:HGNC Symbol;Acc:HGNC:8876]" | 5632 | 0.021 | 0.2132 | No |
| 237 | DLAT | dihydrolipoamide S-acetyltransferase [Source:HGNC Symbol;Acc:HGNC:2896] | 5647 | 0.020 | 0.2123 | No |
| 238 | ADSL | adenylosuccinate lyase [Source:HGNC Symbol;Acc:HGNC:291] | 5685 | 0.017 | 0.2094 | No |
| 239 | BID | BH3 interacting domain death agonist [Source:HGNC Symbol;Acc:HGNC:1050] | 5715 | 0.014 | 0.2072 | No |
| 240 | FH | fumarate hydratase [Source:HGNC Symbol;Acc:HGNC:3700] | 5729 | 0.013 | 0.2063 | No |
| 241 | GYG2 | glycogenin 2 [Source:HGNC Symbol;Acc:HGNC:4700] | 5756 | 0.011 | 0.2043 | No |
| 242 | GALK1 | galactokinase 1 [Source:HGNC Symbol;Acc:HGNC:4118] | 5785 | 0.009 | 0.2021 | No |
| 243 | GPX2 | glutathione peroxidase 2 [Source:HGNC Symbol;Acc:HGNC:4554] | 5808 | 0.008 | 0.2004 | No |
| 244 | AKR1C4 | aldo-keto reductase family 1 member C4 [Source:HGNC Symbol;Acc:HGNC:387] | 5843 | 0.005 | 0.1977 | No |
| 245 | GFPT1 | glutamine--fructose-6-phosphate transaminase 1 [Source:HGNC Symbol;Acc:HGNC:4241] | 5847 | 0.005 | 0.1975 | No |
| 246 | GFPT2 | glutamine-fructose-6-phosphate transaminase 2 [Source:HGNC Symbol;Acc:HGNC:4242] | 5849 | 0.005 | 0.1975 | No |
| 247 | CYB5A | cytochrome b5 type A [Source:HGNC Symbol;Acc:HGNC:2570] | 5850 | 0.005 | 0.1975 | No |
| 248 | POLG2 | "DNA polymerase gamma 2, accessory subunit [Source:HGNC Symbol;Acc:HGNC:9180]" | 5892 | 0.003 | 0.1943 | No |
| 249 | MC4R | melanocortin 4 receptor [Source:HGNC Symbol;Acc:HGNC:6932] | 5918 | 0.001 | 0.1923 | No |
| 250 | CBFA2T3 | CBFA2/RUNX1 partner transcriptional co-repressor 3 [Source:HGNC Symbol;Acc:HGNC:1537] | 5951 | -0.001 | 0.1897 | No |
| 251 | OXA1L | OXA1L mitochondrial inner membrane protein [Source:HGNC Symbol;Acc:HGNC:8526] | 5974 | -0.003 | 0.1880 | No |
| 252 | COX10 | cytochrome c oxidase assembly factor heme A:farnesyltransferase COX10 [Source:HGNC Symbol;Acc:HGNC:2260] | 6092 | -0.012 | 0.1787 | No |
| 253 | ATP5PF | ATP synthase peripheral stalk subunit F6 [Source:HGNC Symbol;Acc:HGNC:847] | 6109 | -0.013 | 0.1775 | No |
| 254 | GLRX2 | glutaredoxin 2 [Source:HGNC Symbol;Acc:HGNC:16065] | 6116 | -0.014 | 0.1771 | No |
| 255 | STBD1 | starch binding domain 1 [Source:HGNC Symbol;Acc:HGNC:24854] | 6139 | -0.015 | 0.1755 | No |
| 256 | EIF6 | eukaryotic translation initiation factor 6 [Source:HGNC Symbol;Acc:HGNC:6159] | 6140 | -0.015 | 0.1756 | No |
| 257 | MCHR1 | melanin concentrating hormone receptor 1 [Source:HGNC Symbol;Acc:HGNC:4479] | 6242 | -0.024 | 0.1677 | No |
| 258 | GYS2 | glycogen synthase 2 [Source:HGNC Symbol;Acc:HGNC:4707] | 6316 | -0.029 | 0.1621 | No |
| 259 | NOP53 | NOP53 ribosome biogenesis factor [Source:HGNC Symbol;Acc:HGNC:4333] | 6334 | -0.031 | 0.1609 | No |
| 260 | ATP5ME | ATP synthase membrane subunit e [Source:HGNC Symbol;Acc:HGNC:846] | 6365 | -0.032 | 0.1588 | No |
| 261 | CYP19A1 | cytochrome P450 family 19 subfamily A member 1 [Source:HGNC Symbol;Acc:HGNC:2594] | 6388 | -0.034 | 0.1573 | No |
| 262 | ACADVL | acyl-CoA dehydrogenase very long chain [Source:HGNC Symbol;Acc:HGNC:92] | 6394 | -0.034 | 0.1571 | No |
| 263 | ATP5F1E | ATP synthase F1 subunit epsilon [Source:HGNC Symbol;Acc:HGNC:838] | 6405 | -0.036 | 0.1566 | No |
| 264 | IFNG | interferon gamma [Source:HGNC Symbol;Acc:HGNC:5438] | 6410 | -0.036 | 0.1565 | No |
| 265 | PHKG1 | phosphorylase kinase catalytic subunit gamma 1 [Source:HGNC Symbol;Acc:HGNC:8930] | 6451 | -0.039 | 0.1536 | No |
| 266 | CYBB | cytochrome b-245 beta chain [Source:HGNC Symbol;Acc:HGNC:2578] | 6610 | -0.050 | 0.1414 | No |
| 267 | IL4 | interleukin 4 [Source:HGNC Symbol;Acc:HGNC:6014] | 6673 | -0.054 | 0.1368 | No |
| 268 | SLC25A23 | solute carrier family 25 member 23 [Source:HGNC Symbol;Acc:HGNC:19375] | 6676 | -0.054 | 0.1370 | No |
| 269 | HK1 | hexokinase 1 [Source:HGNC Symbol;Acc:HGNC:4922] | 6728 | -0.058 | 0.1334 | No |
| 270 | NDUFA5 | NADH:ubiquinone oxidoreductase subunit A5 [Source:HGNC Symbol;Acc:HGNC:7688] | 6789 | -0.062 | 0.1291 | No |
| 271 | NCF2 | neutrophil cytosolic factor 2 [Source:HGNC Symbol;Acc:HGNC:7661] | 6871 | -0.069 | 0.1231 | No |
| 272 | MTFR1 | mitochondrial fission regulator 1 [Source:HGNC Symbol;Acc:HGNC:29510] | 6890 | -0.071 | 0.1222 | No |
| 273 | NR1D1 | nuclear receptor subfamily 1 group D member 1 [Source:HGNC Symbol;Acc:HGNC:7962] | 6974 | -0.078 | 0.1161 | No |
| 274 | GIPR | gastric inhibitory polypeptide receptor [Source:HGNC Symbol;Acc:HGNC:4271] | 6979 | -0.078 | 0.1164 | No |
| 275 | PPP1R1A | protein phosphatase 1 regulatory inhibitor subunit 1A [Source:HGNC Symbol;Acc:HGNC:9286] | 7040 | -0.083 | 0.1122 | No |
| 276 | XYLB | xylulokinase [Source:HGNC Symbol;Acc:HGNC:12839] | 7054 | -0.084 | 0.1118 | No |
| 277 | RB1CC1 | RB1 inducible coiled-coil 1 [Source:HGNC Symbol;Acc:HGNC:15574] | 7056 | -0.084 | 0.1123 | No |
| 278 | G6PD | glucose-6-phosphate dehydrogenase [Source:HGNC Symbol;Acc:HGNC:4057] | 7082 | -0.086 | 0.1110 | No |
| 279 | IDH1 | isocitrate dehydrogenase (NADP(+)) 1 [Source:HGNC Symbol;Acc:HGNC:5382] | 7109 | -0.089 | 0.1096 | No |
| 280 | AVP | arginine vasopressin [Source:HGNC Symbol;Acc:HGNC:894] | 7201 | -0.096 | 0.1030 | No |
| 281 | GPD2 | glycerol-3-phosphate dehydrogenase 2 [Source:HGNC Symbol;Acc:HGNC:4456] | 7206 | -0.097 | 0.1034 | No |
| 282 | GFUS | GDP-L-fucose synthase [Source:HGNC Symbol;Acc:HGNC:12390] | 7317 | -0.103 | 0.0954 | No |
| 283 | GYS1 | glycogen synthase 1 [Source:HGNC Symbol;Acc:HGNC:4706] | 7345 | -0.105 | 0.0940 | No |
| 284 | GPD1 | glycerol-3-phosphate dehydrogenase 1 [Source:HGNC Symbol;Acc:HGNC:4455] | 7420 | -0.112 | 0.0889 | No |
| 285 | ASPH | aspartate beta-hydroxylase [Source:HGNC Symbol;Acc:HGNC:757] | 7533 | -0.121 | 0.0809 | No |
| 286 | ACTN3 | actinin alpha 3 [Source:HGNC Symbol;Acc:HGNC:165] | 7557 | -0.124 | 0.0799 | No |
| 287 | PER2 | period circadian regulator 2 [Source:HGNC Symbol;Acc:HGNC:8846] | 7663 | -0.132 | 0.0725 | No |
| 288 | LOXL2 | lysyl oxidase like 2 [Source:HGNC Symbol;Acc:HGNC:6666] | 7675 | -0.133 | 0.0726 | No |
| 289 | GCGR | glucagon receptor [Source:HGNC Symbol;Acc:HGNC:4192] | 7697 | -0.134 | 0.0719 | No |
| 290 | PIK3CA | "phosphatidylinositol-4,5-bisphosphate 3-kinase catalytic subunit alpha [Source:HGNC Symbol;Acc:HGNC:8975]" | 7733 | -0.136 | 0.0702 | No |
| 291 | IDO1 | "indoleamine 2,3-dioxygenase 1 [Source:HGNC Symbol;Acc:HGNC:6059]" | 7782 | -0.140 | 0.0674 | No |
| 292 | ADIPOQ | "adiponectin, C1Q and collagen domain containing [Source:HGNC Symbol;Acc:HGNC:13633]" | 7792 | -0.141 | 0.0677 | No |
| 293 | ACSS3 | acyl-CoA synthetase short chain family member 3 [Source:HGNC Symbol;Acc:HGNC:24723] | 7821 | -0.143 | 0.0665 | No |
| 294 | APP | amyloid beta precursor protein [Source:HGNC Symbol;Acc:HGNC:620] | 7835 | -0.143 | 0.0666 | No |
| 295 | PHKA1 | phosphorylase kinase regulatory subunit alpha 1 [Source:HGNC Symbol;Acc:HGNC:8925] | 7896 | -0.147 | 0.0629 | No |
| 296 | TRPV4 | transient receptor potential cation channel subfamily V member 4 [Source:HGNC Symbol;Acc:HGNC:18083] | 7925 | -0.149 | 0.0617 | No |
| 297 | NDOR1 | NADPH dependent diflavin oxidoreductase 1 [Source:HGNC Symbol;Acc:HGNC:29838] | 7982 | -0.154 | 0.0584 | No |
| 298 | FXN | frataxin [Source:HGNC Symbol;Acc:HGNC:3951] | 8057 | -0.160 | 0.0537 | No |
| 299 | INPP5K | inositol polyphosphate-5-phosphatase K [Source:HGNC Symbol;Acc:HGNC:33882] | 8103 | -0.163 | 0.0513 | No |
| 300 | ACSM1 | acyl-CoA synthetase medium chain family member 1 [Source:HGNC Symbol;Acc:HGNC:18049] | 8210 | -0.172 | 0.0441 | No |
| 301 | H6PD | hexose-6-phosphate dehydrogenase/glucose 1-dehydrogenase [Source:HGNC Symbol;Acc:HGNC:4795] | 8303 | -0.178 | 0.0381 | No |
| 302 | PDE12 | phosphodiesterase 12 [Source:HGNC Symbol;Acc:HGNC:25386] | 8306 | -0.178 | 0.0392 | No |
| 303 | PHKA2 | phosphorylase kinase regulatory subunit alpha 2 [Source:HGNC Symbol;Acc:HGNC:8926] | 8333 | -0.181 | 0.0385 | No |
| 304 | CYCS | "cytochrome c, somatic [Source:HGNC Symbol;Acc:HGNC:19986]" | 8410 | -0.186 | 0.0338 | No |
| 305 | HDAC4 | histone deacetylase 4 [Source:HGNC Symbol;Acc:HGNC:14063] | 8416 | -0.187 | 0.0348 | No |
| 306 | ALDOB | "aldolase, fructose-bisphosphate B [Source:HGNC Symbol;Acc:HGNC:417]" | 8421 | -0.187 | 0.0359 | No |
| 307 | STAT3 | signal transducer and activator of transcription 3 [Source:HGNC Symbol;Acc:HGNC:11364] | 8453 | -0.190 | 0.0348 | No |
| 308 | LEPR | leptin receptor [Source:HGNC Symbol;Acc:HGNC:6554] | 8479 | -0.192 | 0.0342 | No |
| 309 | OGDH | oxoglutarate dehydrogenase [Source:HGNC Symbol;Acc:HGNC:8124] | 8503 | -0.194 | 0.0339 | No |
| 310 | SORBS1 | sorbin and SH3 domain containing 1 [Source:HGNC Symbol;Acc:HGNC:14565] | 8509 | -0.194 | 0.0349 | No |
| 311 | PGK1 | phosphoglycerate kinase 1 [Source:HGNC Symbol;Acc:HGNC:8896] | 8513 | -0.195 | 0.0361 | No |
| 312 | SIRT6 | sirtuin 6 [Source:HGNC Symbol;Acc:HGNC:14934] | 8532 | -0.196 | 0.0361 | No |
| 313 | RHOA | ras homolog family member A [Source:HGNC Symbol;Acc:HGNC:667] | 8601 | -0.201 | 0.0322 | No |
| 314 | RUBCNL | rubicon like autophagy enhancer [Source:HGNC Symbol;Acc:HGNC:20420] | 8615 | -0.202 | 0.0327 | No |
| 315 | SLC25A22 | solute carrier family 25 member 22 [Source:HGNC Symbol;Acc:HGNC:19954] | 8682 | -0.208 | 0.0289 | No |
| 316 | SLC25A13 | solute carrier family 25 member 13 [Source:HGNC Symbol;Acc:HGNC:10983] | 8713 | -0.210 | 0.0281 | No |
| 317 | UGP2 | UDP-glucose pyrophosphorylase 2 [Source:HGNC Symbol;Acc:HGNC:12527] | 8735 | -0.211 | 0.0280 | No |
| 318 | LEP | leptin [Source:HGNC Symbol;Acc:HGNC:6553] | 8740 | -0.212 | 0.0293 | No |
| 319 | PTGES3 | prostaglandin E synthase 3 [Source:HGNC Symbol;Acc:HGNC:16049] | 8802 | -0.217 | 0.0260 | No |
| 320 | FLCN | folliculin [Source:HGNC Symbol;Acc:HGNC:27310] | 8848 | -0.221 | 0.0241 | No |
| 321 | PRKAA2 | protein kinase AMP-activated catalytic subunit alpha 2 [Source:HGNC Symbol;Acc:HGNC:9377] | 8864 | -0.222 | 0.0245 | No |
| 322 | DEGS1 | "delta 4-desaturase, sphingolipid 1 [Source:HGNC Symbol;Acc:HGNC:13709]" | 8885 | -0.223 | 0.0246 | No |
| 323 | EPM2AIP1 | EPM2A interacting protein 1 [Source:HGNC Symbol;Acc:HGNC:19735] | 8899 | -0.225 | 0.0252 | No |
| 324 | ADH7 | "alcohol dehydrogenase 7 (class IV), mu or sigma polypeptide [Source:HGNC Symbol;Acc:HGNC:256]" | 8906 | -0.225 | 0.0264 | No |
| 325 | ABCD1 | ATP binding cassette subfamily D member 1 [Source:HGNC Symbol;Acc:HGNC:61] | 8930 | -0.226 | 0.0263 | No |
| 326 | ADH1B | "alcohol dehydrogenase 1B (class I), beta polypeptide [Source:HGNC Symbol;Acc:HGNC:250]" | 9036 | -0.235 | 0.0196 | No |
| 327 | PHLDA2 | pleckstrin homology like domain family A member 2 [Source:HGNC Symbol;Acc:HGNC:12385] | 9059 | -0.237 | 0.0196 | No |
| 328 | PDHA2 | pyruvate dehydrogenase E1 subunit alpha 2 [Source:HGNC Symbol;Acc:HGNC:8807] | 9124 | -0.241 | 0.0163 | No |
| 329 | GNMT | glycine N-methyltransferase [Source:HGNC Symbol;Acc:HGNC:4415] | 9208 | -0.248 | 0.0115 | No |
| 330 | GIT1 | GIT ArfGAP 1 [Source:HGNC Symbol;Acc:HGNC:4272] | 9236 | -0.251 | 0.0112 | No |
| 331 | PHGDH | phosphoglycerate dehydrogenase [Source:HGNC Symbol;Acc:HGNC:8923] | 9245 | -0.252 | 0.0125 | No |
| 332 | ADH1A | "alcohol dehydrogenase 1A (class I), alpha polypeptide [Source:HGNC Symbol;Acc:HGNC:249]" | 9283 | -0.254 | 0.0114 | No |
| 333 | ALDH1L1 | aldehyde dehydrogenase 1 family member L1 [Source:HGNC Symbol;Acc:HGNC:3978] | 9302 | -0.256 | 0.0119 | No |
| 334 | DHRS2 | dehydrogenase/reductase 2 [Source:HGNC Symbol;Acc:HGNC:18349] | 9363 | -0.261 | 0.0090 | No |
| 335 | ENTPD5 | ectonucleoside triphosphate diphosphohydrolase 5 (inactive) [Source:HGNC Symbol;Acc:HGNC:3367] | 9414 | -0.265 | 0.0070 | No |
| 336 | ACO1 | aconitase 1 [Source:HGNC Symbol;Acc:HGNC:117] | 9426 | -0.266 | 0.0081 | No |
| 337 | ARNT | aryl hydrocarbon receptor nuclear translocator [Source:HGNC Symbol;Acc:HGNC:700] | 9427 | -0.267 | 0.0101 | No |
| 338 | PRLH | prolactin releasing hormone [Source:HGNC Symbol;Acc:HGNC:17945] | 9443 | -0.268 | 0.0109 | No |
| 339 | ADH6 | alcohol dehydrogenase 6 (class V) [Source:HGNC Symbol;Acc:HGNC:255] | 9504 | -0.274 | 0.0082 | No |
| 340 | ENO1 | enolase 1 [Source:HGNC Symbol;Acc:HGNC:3350] | 9606 | -0.284 | 0.0022 | No |
| 341 | AK4 | adenylate kinase 4 [Source:HGNC Symbol;Acc:HGNC:363] | 9682 | -0.290 | -0.0016 | No |
| 342 | RPIA | ribose 5-phosphate isomerase A [Source:HGNC Symbol;Acc:HGNC:10297] | 9793 | -0.301 | -0.0082 | No |
| 343 | MLXIPL | MLX interacting protein like [Source:HGNC Symbol;Acc:HGNC:12744] | 9882 | -0.310 | -0.0129 | No |
| 344 | RDH16 | retinol dehydrogenase 16 [Source:HGNC Symbol;Acc:HGNC:29674] | 9900 | -0.312 | -0.0119 | No |
| 345 | PPIF | peptidylprolyl isomerase F [Source:HGNC Symbol;Acc:HGNC:9259] | 9999 | -0.321 | -0.0174 | No |
| 346 | HK2 | hexokinase 2 [Source:HGNC Symbol;Acc:HGNC:4923] | 10030 | -0.325 | -0.0174 | No |
| 347 | COQ10B | coenzyme Q10B [Source:HGNC Symbol;Acc:HGNC:25819] | 10099 | -0.331 | -0.0203 | No |
| 348 | PKLR | pyruvate kinase L/R [Source:HGNC Symbol;Acc:HGNC:9020] | 10120 | -0.333 | -0.0195 | No |
| 349 | GCK | glucokinase [Source:HGNC Symbol;Acc:HGNC:4195] | 10137 | -0.335 | -0.0182 | No |
| 350 | POR | cytochrome p450 oxidoreductase [Source:HGNC Symbol;Acc:HGNC:9208] | 10178 | -0.338 | -0.0189 | No |
| 351 | ETFDH | electron transfer flavoprotein dehydrogenase [Source:HGNC Symbol;Acc:HGNC:3483] | 10203 | -0.341 | -0.0183 | No |
| 352 | PRDM16 | PR/SET domain 16 [Source:HGNC Symbol;Acc:HGNC:14000] | 10211 | -0.342 | -0.0163 | No |
| 353 | AACS | acetoacetyl-CoA synthetase [Source:HGNC Symbol;Acc:HGNC:21298] | 10226 | -0.344 | -0.0148 | No |
| 354 | GBE1 | "1,4-alpha-glucan branching enzyme 1 [Source:HGNC Symbol;Acc:HGNC:4180]" | 10356 | -0.357 | -0.0225 | No |
| 355 | PDX1 | pancreatic and duodenal homeobox 1 [Source:HGNC Symbol;Acc:HGNC:6107] | 10409 | -0.362 | -0.0240 | No |
| 356 | IDH3A | isocitrate dehydrogenase (NAD(+)) 3 catalytic subunit alpha [Source:HGNC Symbol;Acc:HGNC:5384] | 10426 | -0.364 | -0.0225 | No |
| 357 | TAFAZZIN | "tafazzin, phospholipid-lysophospholipid transacylase [Source:HGNC Symbol;Acc:HGNC:11577]" | 10429 | -0.364 | -0.0200 | No |
| 358 | PPARGC1A | PPARG coactivator 1 alpha [Source:HGNC Symbol;Acc:HGNC:9237] | 10504 | -0.374 | -0.0231 | No |
| 359 | DLST | dihydrolipoamide S-succinyltransferase [Source:HGNC Symbol;Acc:HGNC:2911] | 10634 | -0.386 | -0.0305 | No |
| 360 | CS | citrate synthase [Source:HGNC Symbol;Acc:HGNC:2422] | 10748 | -0.399 | -0.0366 | No |
| 361 | OGDHL | oxoglutarate dehydrogenase L [Source:HGNC Symbol;Acc:HGNC:25590] | 10754 | -0.399 | -0.0340 | No |
| 362 | PLEC | plectin [Source:HGNC Symbol;Acc:HGNC:9069] | 10799 | -0.405 | -0.0345 | No |
| 363 | DDIT4 | DNA damage inducible transcript 4 [Source:HGNC Symbol;Acc:HGNC:24944] | 10818 | -0.407 | -0.0329 | No |
| 364 | PHKG2 | phosphorylase kinase catalytic subunit gamma 2 [Source:HGNC Symbol;Acc:HGNC:8931] | 10830 | -0.408 | -0.0307 | No |
| 365 | PGD | phosphogluconate dehydrogenase [Source:HGNC Symbol;Acc:HGNC:8891] | 10845 | -0.410 | -0.0288 | No |
| 366 | POMC | proopiomelanocortin [Source:HGNC Symbol;Acc:HGNC:9201] | 11010 | -0.431 | -0.0387 | No |
| 367 | PPARA | peroxisome proliferator activated receptor alpha [Source:HGNC Symbol;Acc:HGNC:9232] | 11110 | -0.445 | -0.0433 | No |
| 368 | LDHA | lactate dehydrogenase A [Source:HGNC Symbol;Acc:HGNC:6535] | 11134 | -0.448 | -0.0418 | No |
| 369 | GSK3B | glycogen synthase kinase 3 beta [Source:HGNC Symbol;Acc:HGNC:4617] | 11157 | -0.451 | -0.0402 | No |
| 370 | HKDC1 | hexokinase domain containing 1 [Source:HGNC Symbol;Acc:HGNC:23302] | 11172 | -0.454 | -0.0379 | No |
| 371 | MYBBP1A | MYB binding protein 1a [Source:HGNC Symbol;Acc:HGNC:7546] | 11188 | -0.456 | -0.0357 | No |
| 372 | GAPDH | glyceraldehyde-3-phosphate dehydrogenase [Source:HGNC Symbol;Acc:HGNC:4141] | 11268 | -0.467 | -0.0386 | No |
| 373 | PPARD | peroxisome proliferator activated receptor delta [Source:HGNC Symbol;Acc:HGNC:9235] | 11301 | -0.471 | -0.0376 | No |
| 374 | IDH3B | isocitrate dehydrogenase (NAD(+)) 3 non-catalytic subunit beta [Source:HGNC Symbol;Acc:HGNC:5385] | 11304 | -0.472 | -0.0342 | No |
| 375 | STEAP4 | STEAP4 metalloreductase [Source:HGNC Symbol;Acc:HGNC:21923] | 11340 | -0.477 | -0.0335 | No |
| 376 | NFATC4 | nuclear factor of activated T cells 4 [Source:HGNC Symbol;Acc:HGNC:7778] | 11363 | -0.481 | -0.0316 | No |
| 377 | IRS2 | insulin receptor substrate 2 [Source:HGNC Symbol;Acc:HGNC:6126] | 11390 | -0.485 | -0.0301 | No |
| 378 | SCO2 | synthesis of cytochrome C oxidase 2 [Source:HGNC Symbol;Acc:HGNC:10604] | 11410 | -0.488 | -0.0280 | No |
| 379 | PRKAA1 | protein kinase AMP-activated catalytic subunit alpha 1 [Source:HGNC Symbol;Acc:HGNC:9376] | 11499 | -0.501 | -0.0313 | No |
| 380 | AOC2 | amine oxidase copper containing 2 [Source:HGNC Symbol;Acc:HGNC:549] | 11550 | -0.508 | -0.0315 | No |
| 381 | ZBTB7A | zinc finger and BTB domain containing 7A [Source:HGNC Symbol;Acc:HGNC:18078] | 11600 | -0.516 | -0.0315 | No |
| 382 | FOXK2 | forkhead box K2 [Source:HGNC Symbol;Acc:HGNC:6036] | 11609 | -0.517 | -0.0283 | No |
| 383 | ADRB3 | adrenoceptor beta 3 [Source:HGNC Symbol;Acc:HGNC:288] | 11691 | -0.531 | -0.0308 | No |
| 384 | CYP1A2 | cytochrome P450 family 1 subfamily A member 2 [Source:HGNC Symbol;Acc:HGNC:2596] | 11719 | -0.536 | -0.0290 | No |
| 385 | PPP1CB | protein phosphatase 1 catalytic subunit beta [Source:HGNC Symbol;Acc:HGNC:9282] | 11737 | -0.539 | -0.0263 | No |
| 386 | EP300 | E1A binding protein p300 [Source:HGNC Symbol;Acc:HGNC:3373] | 11741 | -0.540 | -0.0225 | No |
| 387 | HIF1A | hypoxia inducible factor 1 subunit alpha [Source:HGNC Symbol;Acc:HGNC:4910] | 11833 | -0.556 | -0.0257 | No |
| 388 | PFKFB1 | "6-phosphofructo-2-kinase/fructose-2,6-biphosphatase 1 [Source:HGNC Symbol;Acc:HGNC:8872]" | 11840 | -0.557 | -0.0220 | No |
| 389 | IRS1 | insulin receptor substrate 1 [Source:HGNC Symbol;Acc:HGNC:6125] | 11841 | -0.557 | -0.0178 | No |
| 390 | ADGRF5 | adhesion G protein-coupled receptor F5 [Source:HGNC Symbol;Acc:HGNC:19030] | 11876 | -0.566 | -0.0163 | No |
| 391 | ZBTB20 | zinc finger and BTB domain containing 20 [Source:HGNC Symbol;Acc:HGNC:13503] | 11902 | -0.572 | -0.0140 | No |
| 392 | MTOR | mechanistic target of rapamycin kinase [Source:HGNC Symbol;Acc:HGNC:3942] | 12047 | -0.605 | -0.0210 | No |
| 393 | ALDH4A1 | aldehyde dehydrogenase 4 family member A1 [Source:HGNC Symbol;Acc:HGNC:406] | 12080 | -0.614 | -0.0190 | No |
| 394 | NOS2 | nitric oxide synthase 2 [Source:HGNC Symbol;Acc:HGNC:7873] | 12262 | -0.665 | -0.0285 | No |
| 395 | TKT | transketolase [Source:HGNC Symbol;Acc:HGNC:11834] | 12373 | -0.700 | -0.0321 | No |
| 396 | GSK3A | glycogen synthase kinase 3 alpha [Source:HGNC Symbol;Acc:HGNC:4616] | 12384 | -0.703 | -0.0277 | No |
| 397 | OGT | O-linked N-acetylglucosamine (GlcNAc) transferase [Source:HGNC Symbol;Acc:HGNC:8127] | 12440 | -0.721 | -0.0267 | No |
| 398 | IL6ST | interleukin 6 cytokine family signal transducer [Source:HGNC Symbol;Acc:HGNC:6021] | 12505 | -0.749 | -0.0262 | No |
| 399 | UCN | urocortin [Source:HGNC Symbol;Acc:HGNC:12516] | 12536 | -0.765 | -0.0229 | No |
| 400 | NR4A3 | nuclear receptor subfamily 4 group A member 3 [Source:HGNC Symbol;Acc:HGNC:7982] | 12543 | -0.769 | -0.0176 | No |
| 401 | SOD2 | superoxide dismutase 2 [Source:HGNC Symbol;Acc:HGNC:11180] | 12685 | -0.853 | -0.0225 | No |
| 402 | MT-ND5 | mitochondrially encoded NADH:ubiquinone oxidoreductase core subunit 5 [Source:HGNC Symbol;Acc:HGNC:7461] | 12743 | -0.906 | -0.0203 | No |
| 403 | AVPR1A | arginine vasopressin receptor 1A [Source:HGNC Symbol;Acc:HGNC:895] | 12751 | -0.913 | -0.0140 | No |
| 404 | MYC | "MYC proto-oncogene, bHLH transcription factor [Source:HGNC Symbol;Acc:HGNC:7553]" | 12801 | -0.970 | -0.0107 | No |
| 405 | INSR | insulin receptor [Source:HGNC Symbol;Acc:HGNC:6091] | 12856 | -1.071 | -0.0070 | No |
| 406 | AKT2 | AKT serine/threonine kinase 2 [Source:HGNC Symbol;Acc:HGNC:392] | 12896 | -1.404 | 0.0003 | No |
Table: GSEA details [plain text format]

  

Fig 2: GOBP\_GENERATION\_OF\_PRECURSOR\_METABOLITES\_AND\_ENERGY      
 Blue-Pink O' Gram in the Space of the Analyzed GeneSet

  

Fig 3: GOBP\_GENERATION\_OF\_PRECURSOR\_METABOLITES\_AND\_ENERGY: Random ES distribution      
 Gene set null distribution of ES for **GOBP\_GENERATION\_OF\_PRECURSOR\_METABOLITES\_AND\_ENERGY**
